# Supplementary figures and images for: Online assessment of medical students’ communication competence in patient encounters: Validation of the VA-MeCo situational judgement test
Source: PLoS One. 2025 Sep 23;20(9):e0332957. doi: 10.1371/journal.pone.0332957 (PMC12456786; doi:10.1371/journal.pone.0332957)

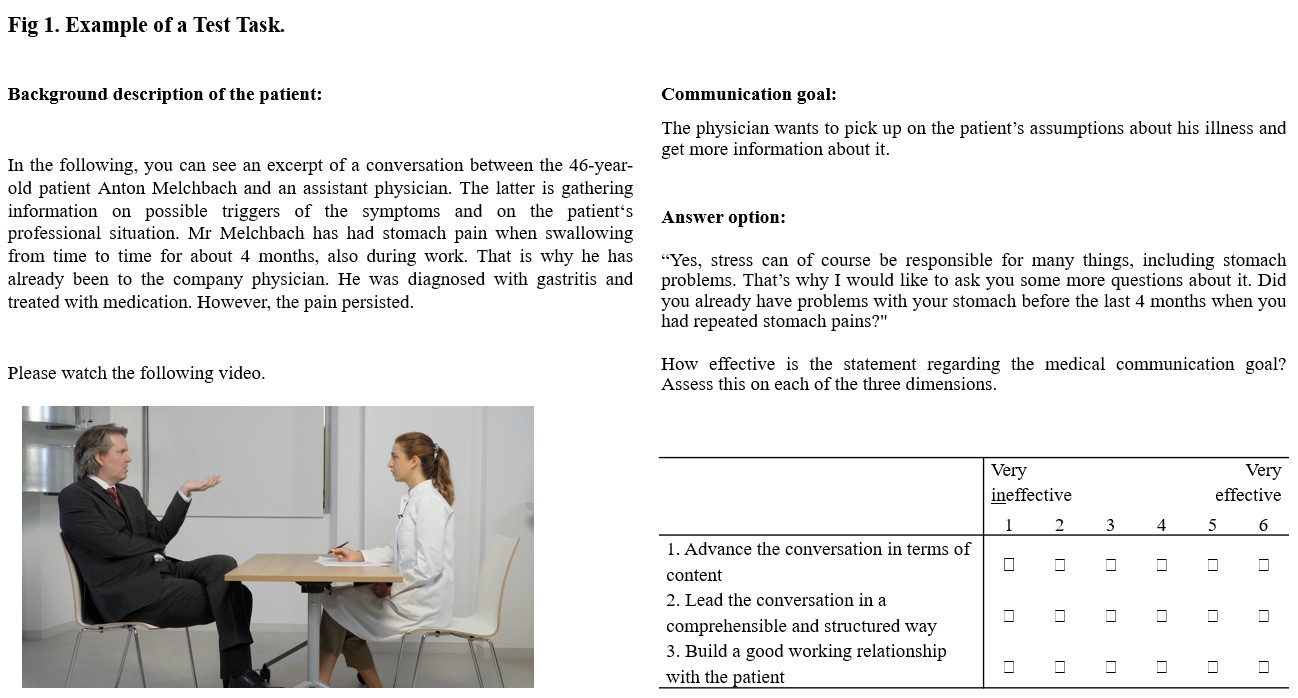

Supplement: S1 Fig — (TIF) [file pone.0332957.s001.tif]

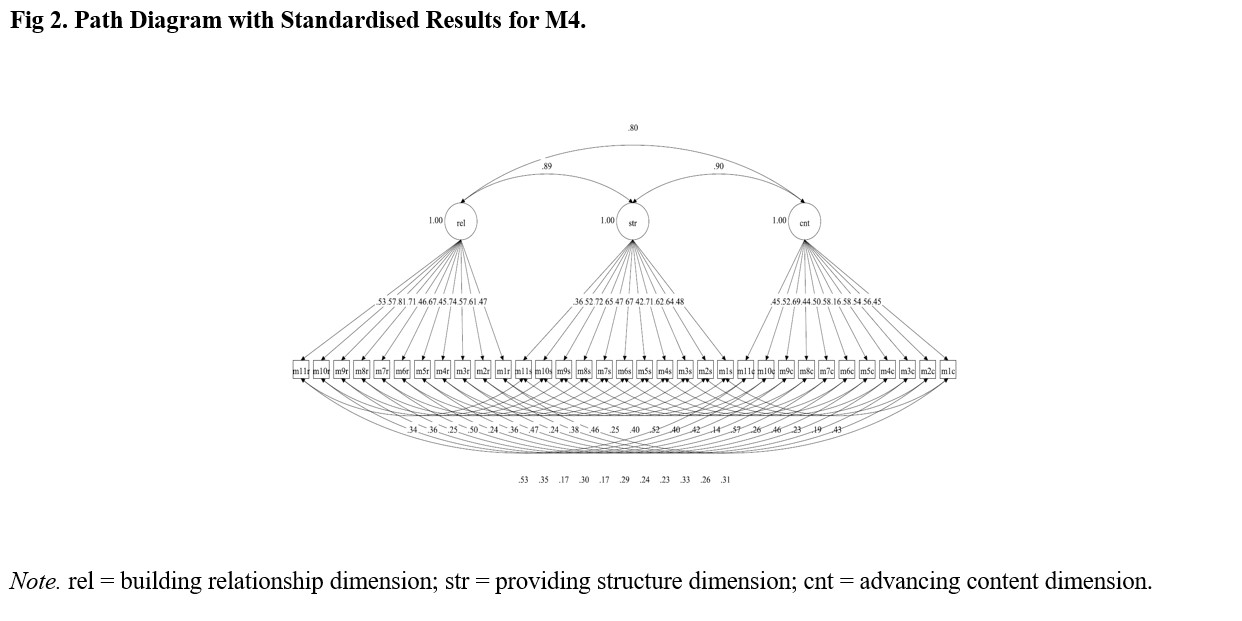

Supplement: S2 Fig — (TIF) [file pone.0332957.s002.tif]
