# Supplementary material for: Online assessment of medical students’ communication competence in patient encounters: Validation of the VA-MeCo situational judgement test
Source: PLoS One. 2025 Sep 23;20(9):e0332957. doi: 10.1371/journal.pone.0332957 (PMC12456786; doi:10.1371/journal.pone.0332957)
Supplement: S1 Table — (DOCX) [file pone.0332957.s003.docx]

**Table S1. Details of the Instruments Used for Measuring Validation Variables.**

| Variable | Instrument name/ reference | Additional information | Cronbach’s α^a^ |
| --- | --- | --- | --- |
| *Cognitive variables* |  |  |  |
| Prior knowledge and  experience | [63] | 7 items on completed learning opportunities and knowledge about medical communication (per item: 0 = *not stated*, 1 = *stated*) | — |
| Study progress | [63] | Semester of study in the medical education program (single item) | — |
| Intelligence | Design a Matrix – Advanced Plus Test (DESIGMA A+) [64] | Intelligence test for differentiating in the upper intelligence range; 31 items | .91 |
| *Patient-interaction variables* | | |  |
| Empathy | Interpersonal Reactivity Index (IRI) [65] | |  |
|  |  | Sub-scale *perspective-taking* (7 items) | .77^b^ |
|  |  | Sub-scale *empathic concern* (7 items) | .71^b^ |
|  |  | Answer format: 1 = *does not describe me well* to 5 = *describes me well* |  |
| Patient orientation | Patient-Provider Orientation Scale (PPOS-D12) [66] | |  |
|  |  | Sub-scale *sharing* (6 items) | .64 |
|  |  | Sub-scale *caring* (6 items) | .67 |
|  |  | Answer format: 1 = *strongly disagree* to 6 = *strongly agree* |  |
| *General personality traits* | | |  |
| Social competence | Inventory of Social Competences – Short Version (ISK-K) [67] | |  |
|  |  | Sub-scale *social orientation* (10 items) | .75 |
|  |  | Sub-scale *offensiveness* (8 items) | .69 |
|  |  | Sub-scale *self-organisation* (8 items) | .74 |
|  |  | Sub-scale *reflexibility* (7 items) | .70 |
|  |  | Answer format: 1 = *does not apply* at all to 4 = *applies very much* |  |
| Personality | Big Five Inventory – Short Version (BFI-K) [68] | |  |
|  |  | Sub-scale *extraversion* (4 items) | .81 |
|  |  | Sub-scale *agreeableness* (4 items) | .59 |
|  |  | Sub-scale *conscientiousness* (4 items) | .69 |
|  |  | Sub-scale *neuroticism* (4 items) | .77 |
|  |  | Sub-scale *openness* (5 items) | .70 |
|  |  | Answer format: 1 = *disagree strongly* to 5 = *agree strongly* |  |

*Note*.

^a^Values of Cronbach’s alpha correspond to the documented reliabilities of the instruments. For details, refer to the referenced publications and test documentations.

^b^Standardised Cronbach’s alpha, means of Cronbach’s alpha for males and females.
