# Supplementary material for: Online assessment of medical students’ communication competence in patient encounters: Validation of the VA-MeCo situational judgement test
Source: PLoS One. 2025 Sep 23;20(9):e0332957. doi: 10.1371/journal.pone.0332957 (PMC12456786; doi:10.1371/journal.pone.0332957)
Supplement: S2 Table — (DOCX) [file pone.0332957.s004.docx]

**Table S2.** **Standardised Factor Loadings and Factor Correlations of the Final Three-dimensional CFA-Model (M4).**

| Task | Content | Structure | Relationship |
| --- | --- | --- | --- |
| *Standardised factor loadings* | | | |
| 1 | .47 | .49 | .45 |
| 2 | .54 | .67 | .61 |
| 3 | .60 | .66 | .52 |
| 4 | .56 | .70 | .77 |
| 5 | .17 | .38 | .47 |
| 6 | .50 | .65 | .71 |
| 7 | .50 | .45 | .47 |
| 8 | .44 | .63 | .74 |
| 9 | .69 | .74 | .80 |
| 10 | .54 | .50 | .55 |
| 11 | .55 | .36 | .45 |
| *Factor correlations* |  |  |  |
|  | Content | Structure | Relationship |
| Content | — |  |  |
| Structure | .90 | — |  |
| Relationship | .80 | .89 | — |

*Note*. All factor loadings and correlations are statistically significant.
