# Supplementary material for: Online assessment of medical students’ communication competence in patient encounters: Validation of the VA-MeCo situational judgement test
Source: PLoS One. 2025 Sep 23;20(9):e0332957. doi: 10.1371/journal.pone.0332957 (PMC12456786; doi:10.1371/journal.pone.0332957)
Supplement: S3 Table — (DOCX) [file pone.0332957.s005.docx]

**Table S3.** **Model Fit of Alternative One- and Two-dimensional CFA Models Compared to the Three-dimensional Target Model of MCC (M4).**

| Model | χ^2^-test/Δχ^2^-test | RMSEA  [90% CI] | CFI | SRMR | AIC | BIC | saBIC |
| --- | --- | --- | --- | --- | --- | --- | --- |
| *Three-dimensional model* | | | | |  |  |  |
| M4 Three MCC factors | χ^2^(459) = 733.697, *p* < .001 | .039 [.034, .044] | .921 | .065 | **38770.611** | **39307.760** | **38879.405** |
| *Two-dimensional models* | | | | |  |  |  |
| M5 General factor | χ^2^(462) = 867.470, *p* < .001 | .047 [.042, .052] | .883 | .066 | 38930.855 | 39456.068 | 39037.232 |
| M6 Content, other^a^ | χ^2^(461) = 806.843, *p* < .001 | .044 [.039, .049] | .900 | .065 | 38858.283 | 39387.475 | 38965.466 |
| M7 Structure, other^b^ | χ^2^(461) = 837.609, *p* < .001 | .045 [.041, .050] | .891 | .067 | 38892.535 | 39421.726 | 38999.717 |
| M8 Relationship, other^c^ | χ^2^(461) = 776.440, *p* < .001 | .042 [.036, .047] | .909 | .066 | 38821.458 | 39350.650 | 38928.640 |
| χ2*-difference tests for comparisons of M4 with alternative models* | | | | | | | |
| M4 vs. M5 | Δχ^2^(3) = 90.703, *p* < .001 |  |  |  |  |  |  |
| M4 vs. M6 | Δχ^2^(2) = 46.355, *p* < .001 |  |  |  |  |  |  |
| M4 vs. M7 | Δχ^2^(2) = 116.772, *p* < .001 |  |  |  |  |  |  |
| M4 vs. M8 | Δχ^2^(2) = 26.778, *p* < .001 |  |  |  |  |  |  |

*Notes*. Δχ^2^ = scaled χ^2^-difference for robust maximum likelihood estimation, RMSEA = root mean square error of approximation; CFI = comparative fit index; SRMR = standardised root mean square residual; AIC = Akaike information criterion; BIC = Bayesian information criterion; saBIC = sample size adjusted BIC; CI = confidence interval; boldface = lowest values for information criteria.

^a^ other = merging of structure and relationship sub-scales.

^b^ other = merging of content and relationship sub-scales.

^c^ other = merging of content and structure sub-scales.
