# Supplementary material for: Online assessment of medical students’ communication competence in patient encounters: Validation of the VA-MeCo situational judgement test
Source: PLoS One. 2025 Sep 23;20(9):e0332957. doi: 10.1371/journal.pone.0332957 (PMC12456786; doi:10.1371/journal.pone.0332957)
Supplement: Attachment — Submitted filename: Reiser_etal_Response to Reviewers.docx [file pone.0332957.s007.docx]

**Revision on the article: Online assessment of medical students’ communication competence in patient encounters: Validation of the VA-MeCo situational judgement test (Manuscript number** **PONE-D-25-19255)**

Dear referees:

We thank the editor and the reviewers very much for their helpful comments and recommendations on our manuscript. We believe the manuscript has profited substantially from them.

Below, we provide a detailed account of the revisions we made. For enhancing clarity, we printed the original reviewers’ comments in bold, our responses in italics and quotations from new or revised passages in plain text. In the manuscript we used Word’s track changes feature to highlight the changes we made.

We hope we have addressed the editor’s and reviewers’ concerns adequately and that the referees judge the revised article suitable for publication in PLOS ONE.

Sincerely,

the authors

**Listing of reviewers’ comments and authors’ responses**

**************************

***Comments by the Reviewers***

**************************

**Comments by Reviewer I**

**RI.1:**

**As the authors identify, professionalism and in particular communication skills are some of the most important and yet notoriously difficult skills to teach to medical students.**

**Perhaps the first step to teaching or learning anything is to define what it is that we are attempting to teach or learn. This study goes a long way towards clarifying what it is we mean when we say "Medical Communication Competence".**

**Any step which bringings us closer to the development of a means to accurately measure these skills without bias provides feedback that can be used to improve students' communication skills and teachers' ability to teach these skills. By validating the constructs that underlie MCC this study supports the future development of more scaffolded approaches to teaching and learning MCC.**

*Authors’ Response: Thank you very much for this motivating comment!*

**RI.2:**

**I would note that the article was information dense and that it took me several reads to unpack the study itself and the conclusions that the authors' had drawn. I would suggest the incorporation of simple visual aids to assist readers to understand the core concepts more easily... Perhaps Radar Diagrams or the like showing the factors and their correlation coefficients might be useful.**

*Authors’ Response: Thank you for this valuable comment. We recognise that the information presented is dense. In response to your suggestion, we have added a heatmap for the Pearson correlations of the measures with the three dimensions of MCC to better visualise parts of the main content and improve clarity. We hope this makes the information more accessible and easier to understand.*

Text [Fig 1]:

**Fig 1. Heatmap for Pearson Correlations of the Measures with the Three Dimensions of MCC.**

*Notes*. The correlations in boldface are in line with the stated hypotheses (see Table 1). * = p ≤ .05 (two-tailed). ** = p ≤ .01 (two-tailed).

**Comments by Reviewer II**

**RII.1:**

**In the background include a paragraph that addresses what is currently happening with the measurement of communication competence in health sciences students, that is, whether current teaching methods and/or didactis are effective or why they are a challenge, where they are failing, or why current methods are not working.**

*Authors’ Response: We thank the reviewer for this valuable suggestion and have revised the Background section accordingly. The revised text now briefly outlines current approaches to assessing medical communication competence (MCC), including objective structured clinical examinations [1] and multiple mini interviews [2] with standardised patients and checklists. Such methods are often considered gold standards in teaching and assessment [1,3]; however, they are resource-intensive, requiring, for example, the development of valid rating scales [4]**. In addition, available MCC assessment instruments differ in focus and in the aspects of communication competence they capture, which limits comparability and standardisation [5]. For these reasons, situational judgement tests (SJTs) have received increasing attention. Research indicates that SJTs have favourable psychometric properties in the assessment of constructs relevant for medical education [6,7]. Moreover, they assess individuals’ abilities to provide knowledge-based and situation-specific judgements in authentic scenarios [text p. 6, lines 114 to 115]. Please see also our response to the following comment (R2.II).*

Text [p. 5, lines 84 to 90]: Standardised patient encounters in objective structured clinical examinations (OSCEs) [1] or multiple mini interviews [3] are frequently used and often considered the gold standard in teaching and assessment of MCC [1,3]. However, they are particularly resource-intensive to design and implement. In addition, available MCC assessment instruments differ in focus and in the aspects of communication competence they capture, which limits comparability and standardisation. To address such challenges, there is growing interest in using situational judgement tests (SJTs) as assessment tools for a wide range of competences [8].

**RII.2:**

**Also, justify why a scale or test allows for the measurement of communication competence, which is an aspect that requires procedural and attitudinal skills, not just cognitive skills or decision-making or judgment.**

*Authors’ Response: Thank you for highlighting this important point. We fully agree that a comprehensive assessment of MCC should encompass not only cognitive but also procedural and attitudinal components. However, no single instrument can provide an in-depth evaluation of all these dimensions simultaneously. Indeed, research on competence assessment has even called for distinguishing assessments of the cognitive foundations of competent performance from motivational and attitudinal factors [9,10]. A particular strength of the SJT-format is its ability to assess an individual’s capacity to make knowledge-based, situation-specific judgements in authentic scenarios [text p. 6, lines 114 to 115]. According to theoretical frameworks, such as the competence-as-continuum model [11], these situation-specific skills constitute essential mediators between individual dispositions (e.g. knowledge, motivation, attitudes) and observable performance. In this sense, SJTs may serve as a bridge between purely knowledge-focused measures of MCC and performance-based approaches, such as OSCEs, thereby indirectly capturing procedural knowledge and attitudinal dispositions [11]. While a comprehensive assessment will likely require a combination of instruments, SJTs contribute an important complementary perspective and thereby add value to the measurement of MCC. We have now made this explicit in the Background section, but only briefly to avoid overwhelming readers with theoretical detail.*

Text [p. 7, lines 130 to 134]: SJTs can therefore help bridge the gap between knowledge-based measures of MCC and behavioural assessments, such as OSCEs. By requiring judgements grounded in authentic scenarios, they indirectly capture procedural knowledge and attitudinal dispositions (e.g. empathy, respect, professionalism), thereby contributing to a more comprehensive evaluation of MCC [11].

**RII.3:**

**Improve the wording of lines 84 and 92, as it currently appears contradictory. First, they state that there is a growing interest in the use of situational judgment tests (SJTs), and then in line 92, they mention "still only a few SJTs."**

*Authors’ Response: Thank you for pointing this out. We have clarified that there is interest in SJTs as assessment tools in medical education more broadly, given their potential to capture a wide range of competences. However, only a small number of SJTs have so far been developed specifically for the measurement of MCC.*

Text [p. 5, lines 89 to 90]: To address such challenges, there is growing interest in using situational judgement tests (SJTs) as assessment tools for a wide range of competences [8].

Text [p. 6, lines 97 to 99]: Despite this growing interest, the number of SJTs developed specifically to assess MCC in medical education remains very limited, and most currently provide only preliminary evidence for their validity.

**RII.4:**

**Methodology: It is suggested included the number of students who did not participate and indicated the reasons.**

*Authors’ Response: Thanks for this comment. Because recruitment was conducted through multiple partner institutions and various channels (e.g. lectures or email lists), it is impossible to determine how many students chose not to participate or their reasons for doing so. Therefore, this information could not be included in the manuscript. We now acknowledge this in the Participants section.*

Text [p. 15, lines 294 to 295]: Note that due to the decentralised recruitment strategy, the amount and reasons for non-participation could not be determined.

**RII.5:**

**Methodology: Please clarify if the 45 minutes were spent administering only the Va-MeCo or all the tests. Please also indicate how many questions were included in the total for all the tests, and what the total number of questions was. Although the authors mention that the information is reviewed according to references, please also mention if everything was self-administered or if there was a tutor who guided them through all the tests, or only the Va-MeCo.**

**All this information is necessary to allow other appropriately trained researchers to fully replicate your study.**

*Authors’ Response: Thanks for this helpful comment. First, the 45 minutes refer specifically to the administration of the VA-MeCo. Exact time records for the additional* *questionnaires and achievement tests are not available because these measures were administered by a commercial psychological testing service that did not provide time stamps. However, a pilot study indicated that completing the entire study (i.e. VA-MeCo plus validation measures) required approximately 1.5 hours on average. This information has now been added to the manuscript.*

*Second, full details on the number and content of all items are already provided in the publicly available codebook and open data (see p.26, lines 560 to 565).*

*Third, participants completed the VA-MeCo, the questionnaires and the achievement tests entirely online. They received written instructions at the start of the study and were guided through the procedure by the survey software, without personal assistance from a tutor. This has now been clarified in the manuscript.*

Text [p. 15, lines 290 to 291]: Participation took approximately 1.5 hours on average, with an average of 45 minutes for completing the VA-MeCo.

Text [p. 15, lines 307 to 309]: The study was conducted individually online: participants received written instructions at the beginning and then were guided through the procedure by the survey software, without personal assistance.

**RII.6:**

**Results: This section provides part of the discussion, as it not only describes the results but also briefly analyzes them. This is permitted by the journal; however, it is suggested that you create a combined Results/Discussion section (commonly labeled "Results and Discussion") or leave it as results only without providing additional information or expressing your opinion.**

*Authors’ Response: We carefully considered the reviewer’s suggestion to restructure this section as either a combined 'Results and Discussion' or 'Results' section. However, we found that omitting the additional contextual information made the findings more difficult for readers to follow and fully understand. For this reason, we have decided to retain the current structure, as we believe it supports clarity and readability.*

**RII.7:**

**Discussion: Include references between lines 385 and 407 that support or contrast your results. For example, it is not clear why it allows for better feedback, or how it relates to feedback. Evidence that supports this or mentions its importance in the process is missing.**

*Authors’ Response: We appreciate the reviewer’s suggestion and have included supporting literature in the revised manuscript.*

**RII.8:**

**Limitations/Conclusion: Include in one of these sections, that communication competency is a procedural and attitudinal competency where students must verbalize and manage their emotions to empathize. Therefore, the current proposal is a cognitive approach, since the interaction of these facets requires further research and other types of instruments/methodologies for its measurement.**

*Authors’ Response: Thank you for this helpful comment. We have included this point to the Limitations section.*

Text [p. 25, lines 518 to 522]: Furthermore, as the VA-MeCo primarily addresses cognitive aspects, further research is needed to examine how these interact with procedural and attitudinal dimensions of MCC (such as verbalising and regulating emotions to convey empathy) and develop combined approaches that capture these facets more directly.

**Comments by Reviewer III**

**RIII.1:**

**This manuscript „Assessment of medical communication competence“ presents a carefully conducted empirical study. The strengths of the manuscript lie in its conceptual clarity, the systematic approach to empirical analysis. It is well formulated in terms of language.**

**The authors describe an empirical analysis of the factorial and construct validity of an online situational judgement test (VA-MeCo), measuring medical students´ communication competence. They provide a detailed and well-structured background on the test and its conceptual foundations. The research question is clearly defined, and the hypotheses are quantified based on empirical findings. The methodology of confirmatory factor analysis seems appropriately selected and transparently described. Construct validity is operationalised and tested using convergent and discriminant variables. The discussion section effectively integrates the results with relevant literature and outlines implications in a differentiated manner.**

*Authors’ Response: Thank you for this motivating feedback.*

**RIII.2:**

**However, the conclusion section stands out from the rest of the manuscript in terms of focus and content. Rather than providing a concise summary of the study’s aims, methods, and main findings, it introduces new aspects that have not been sufficiently addressed in earlier sections. While these points appear clearly relevant and valuable, they would benefit from being integrated into the introduction and discussion to ensure a coherent overall argument and to support the internal consistency of the manuscript.**

*Authors’ Response: We thank the reviewer for raising this important point. We believe the concern arises from our inclusion of practical implications within the Conclusions section. To avoid potential confusion, we have renamed this section ‘Practical implications’ to clarify that its purpose is not to summarise the main study’s central aims and findings, but to outline possible practical implications for medical education. We have carefully considered the reviewer’s suggestion to foreshadow these points earlier in the manuscript. However, we concluded that doing so would overly disrupt the flow of argument in the introduction. We hope that this revision provides an acceptable solution.*

**RIII.3:**

**The study addresses a topic of clear relevance within the field. It enriches the ongoing academic discourse and opens avenues for further research. Overall, the manuscript represents a valuable scholarly contribution and demonstrates a high standard of academic rigor.**

*Authors’ Response: Thank you for this kind and valuable feedback.*

**Comments by Reviewer IV**

**RIV.1:**

**The authors report a rigorous extension of the characterization of an instrument to measure an individual's skills in communication with a patient. The manuscript significantly expands the characterization beyond previous reports.**

*Authors’ Response: Thank you for this encouraging comment!*

**RIV.2:**

**The article would have more impact if it reported changes in learners' communication skills in response to an intervention.**

*Authors’ Response: Thank you for your valuable comment. While the present study focused on instrument development rather than testing an intervention, validated instruments are essential for evaluating the effectiveness of intervention approaches. We therefore believe that our work provides an important foundation for future research on enhancing learners’ communication skills.*

**RIV.3:**

**In terms of the quality of the validation, two tasks (tasks 1 and 5) fail to show standardized factor loadings above 0.5 (a commonly used cut-off) for any of the three subscales.**

*Authors’ Response: We acknowledge the reviewer’s concern regarding the loadings of Items 1 and 5. However, in the psychometric literature, there is no universally accepted threshold for factor loadings, as cut-off values inevitably involve some arbitrariness and are context dependent [12–14]. The threshold of ~ .40 or larger for standardised factor loadings that we applied in our study is frequently recommended in the literature [14–16], although more liberal (≥ .30) or more stringent (≥ .50, as noted by the reviewer) criteria have also been suggested [12,13,17,18]. Importantly, factor solutions should not be judged solely by individual loadings, but rather the overall pattern of loadings, additional statistical indices, and substantive considerations [12,14,16,19].*

*In our study, alongside adequate model fit, each factor was defined by a majority of items with loadings that are substantial by any of the above standards. The overall factor solution is interpretable, the constructs remain theoretically coherent, and the resulting scales demonstrate high reliability. For these reasons, we consider the solution adequately saturated and statistically defensible, while recognising that Item 5 in particular may warrant refinement in future research. We decided to retain Items 1 and 5 because each represents an important step in medical communication and removing them would compromise the instrument’s content validity. Nonetheless, in response to the reviewer’s concern, we have explicitly acknowledged in the Limitations and future research section that some tasks exhibit relatively low factor loadings.*

Text [p. 23, lines 479 to 484]: First, regarding factorial validity, some tasks (particularly Item 5) showed relatively low factor loadings. We retained these items because they represent important steps in medical communication and excluding them would have compromised the instrument’s content validity. Nonetheless, as the overall factor solution is well-defined, theoretically coherent, and yields reliable scales, we consider it defensible, while noting that these tasks may warrant refinement in future research.

**Comments by Reviewer V**

**RV.1:**

**Reviewer comment and suggestions**

**This study on the Video-Based Assessment of Medical Communication Competence (VA-MeCo) presents valuable insights into measuring medical communication competence (MCC) among medical students. However few issues need improvement.**

**Strengths:**

**• Relevance of the Topic: The study addresses a critical area in medical education, emphasizing the importance of effective communication in patient encounters, which is well-documented in literature.**

**• Methodological Rigor: The use of confirmatory factor analysis (CFA) to assess factorial validity adds a strong methodological foundation, providing statistical support for the three-dimensional structure of MCC.**

**• Reliability: The high McDonald’s Ω values indicate that the VA-MeCo test is reliable, reinforcing its potential utility in educational settings.**

**• Construct Validity Exploration: Evaluating both convergent and discriminant validity enhances the understanding of how MCC relates to other cognitive and personality traits, suggesting that the VA-MeCo measures a specific and relevant construct.**

**• Implications for Medical Education: The findings support the integration of structured assessments like VA-MeCo into medical curricula, highlighting their role in improving the competence of future physicians.**

*Authors’ Response: Thank you very much for your detailed and supportive feedback, which we greatly appreciate.*

**RV.2:**

**Areas for Improvement:**

**Sample Diversity: Although the sample size of 395 is substantial, the study should discuss the demographic diversity of participants. If the sample lacks variety in terms of age, gender, ethnicity, or educational background, this may limit the generalizability of the findings.**

*Authors’ Response: Thank you for this valuable comment. We have added further descriptive information on participants’ demographic characteristics to indicate the sample’s coverage. For privacy reasons, the collection of socio-demographic information was limited, and we did not collect data on ethnicity. All participants had equivalent educational backgrounds, as entry to medical education in Germany is highly regulated. We hope this addresses the reviewer’s request.*

Text [p. 15, lines 296 to 301]: The final sample consisted of *N* = 395 medical students, of whom 70.33% were female, 29.04% male, and 0.63% diverse. This gender distribution aligns with official statistics for German medical students [20]. The sample covered all phases of German medical education, albeit with a focus on the pre-clinical and clinical phases where medical communication courses predominantly take place (age: *M* = 23.47, *SD* = 3.86; semester of study: *M* = 5.77, *SD* = 3.55; stage of study: 41.12% pre-clinic, 51.60% clinic, 6.57% practical year, 0.71% other).

**RV.3:**

**Areas for Improvement:**

**Exploration of Deviation in Correlations: While the study mentions that correlation sizes partly deviated from expectations, it does not thoroughly explore the implications of this finding. A deeper analysis could provide insights into why certain relationships were weaker than anticipated and how this might inform future research.**

*Authors’ Response: Thanks for this comment. We agree that deviations in correlation sizes raise interesting questions. However, based on the available data, interpreting the causes of these variations would be largely speculative, which we would prefer to avoid. Possible explanations could include chance fluctuations, content-related factors, methodological aspects, or participant characteristics. Determining whether such deviations replicate and generalise across different validation measures remains an issue for further research. We now explicitly acknowledge this in the Limitations section.*

Text [p. 24, lines 505 to 507]: Moreover, the present data do not allow us to explore the causes of deviations in correlation sizes from the expected values. Future research should investigate whether such patterns replicate across different validation measures.

**RV.4:**

**Areas for Improvement:**

**Longitudinal Validation: The study recommends further validation, but it would strengthen its claims to propose specific longitudinal or cross-validation studies. Demonstrating stability of MCC over time and its predictive validity concerning actual patient interactions would be beneficial.**

*Authors’ Response: Thank you for these recommendations. We have included them as directions for further research.*

Text [p. 25, lines 523 to 528]: Additionally, the stability of MCC over time and its sensitivity to instructional interventions should be investigated. Longitudinal research could assess medical students with the VA-MeCo at multiple points during their studies to track changes in MCC and evaluate the instrument’s sensitivity to communication training. Moreover, examining external and predictive validity by testing associations with performance in both simulated and real patient interactions would further strengthen the instrument.

**RV.5:**

**Areas for Improvement:**

**Practical Application: Though the study indicates that VA-MeCo is useful for assessing MCC, it could provide more detailed recommendations on how the findings can be implemented in medical training programs. For example, how should educators modify curricula based on VA-MeCo results?**

*Authors’ Response: Thank you for this suggestion. We outline practical implications in the section at the end of the paper, however, shortly, as an instrument development study can only partly provide evidence-based recommendations.* *This notwithstanding, because of the close alignment of the VA-MeCo’s tasks with established curricular standards for medical communication training (see p. 7, lines 137 to 139), educators might use test tasks as blueprint for learning tasks in communication training and consider when and where curricula address specific aspects of medical communication. Moreover, test results might be useful to reflect on how curricula currently support students’ development in different dimensions of MCC. This means reviewing whether there are enough learning opportunities for the various content aspects of MCC.*

Text [p. 26, lines 544 to 554]: The present study adds to the existing evidence for the validity of the VA-MeCo in measuring medical students’ MCC in patient encounters. Due to its digital implementation and ease of use, the test can be administered effectively to large groups in medical education. The VA-MeCo is versatile and can be used for both assessment and teaching MCC. In the classroom, faculty can use the test to monitor student progress, provide feedback, and raise awareness of important medical communication issues. In many teaching scenarios, the VA-MeCo can be effectively combined with more resource-intensive methods, such as OSCEs and standardised patients, at different stages of the curriculum. Test results can provide valuable feedback to faculty, supporting curricular quality development. Finally, given its close alignment with standards for medical communication training [21,22], the VA-MeCo’s materials may serve as inspiration for creating new learning opportunities or refining existing ones.

**RV.6:**

**Areas for Improvement:**

**Potential Confounders: While the study suggests that VA-MeCo isn't confounded by traits like intelligence, it could be worthwhile to explore other potential confounding variables that may influence MCC and how these were controlled in the study.**

*Authors’ Response: We agree with the reviewer that it would be valuable to consider additional potential confounding variables and now highlight this as an issue for further research. In addition to the discriminant measures investigated in the present study, we already outlined why the VA-MeCo’s design helps mitigate social desirability bias (see p. 25, lines 529 to 539). Confounding with intelligence and social desirability bias are often regarded as primary concerns for SJT-methodology [23]. Beyond these, we now emphasise that group-related test biases (for instance, differential item functioning by gender or ethnic background) should also be investigated in future research. Addressing these issues was beyond the scope of the present study, as the necessary methodology (e.g. multi-group CFA) would require substantially larger subgroup sample sizes.*

Text [p. 26, lines 539 to 542]: Nonetheless, future research should also examine other potential threats to validity, such as group-related biases (e.g. differential item functioning by gender or ethnic background). Investigating such aspects was beyond the scope of the present study due to the sample size requirements.

**RV.7:**

**NB:**

**Generally, this study is a strong contribution to the field of medical education, offering a rigorously validated instrument for assessing communication skills. Addressing the areas for improvement could enhance its robustness and applicability further, making it a more powerful tool for educators seeking to improve medical communication competence among students.**

*Authors’ Response: Thank you very much for your positive and encouraging feedback. We have carefully considered the suggested areas for improvement and implemented them wherever possible to further strengthen the manuscript.*

**References** (cited in this letter)

1. Cömert M, Zill JM, Christalle E, Dirmaier J, Härter M, Scholl I. Assessing communication skills of medical students in objective structured clinical examinations (OSCE) – a systematic review of rating scales. PLoS ONE. 2016;11:e0152717. doi: 10.1371/journal.pone.0152717 PMID: 27031506.
2. Wolcott MD, Zeeman JM, Cox WC, McLaughlin JE. Using the multiple mini interview as an assessment strategy within the first year of a health professions curriculum. BMC Med Educ. 2018;18:92. doi: 10.1186/s12909-018-1203-5 PMID: 29724211.
3. Lane C, Rollnick S. The use of simulated patients and role-play in communication skills training: a review of the literature to August 2005. Patient Educ Couns. 2007;67:13–20. doi: 10.1016/j.pec.2007.02.011 PMID: 17493780.
4. Schwartzman E, Hsu DI, Law AV, Chung EP. Assessment of patient communication skills during OSCE: examining effectiveness of a training program in minimizing inter-grader variability. Patient Educ Couns. 2011;83:472–7. doi: 10.1016/j.pec.2011.04.001 PMID: 21555198.
5. Reiser S, Schacht L, Thomm E, Figalist C, Janssen L, Schick K, et al. A video-based situational judgement test of medical students' communication competence in patient encounters: development and first evaluation. Patient Educ Couns. 2022;105(5):1283–9. doi: 10.1016/j.pec.2021.08.020 PMID: 34481676. PMID: 34481676.
6. Husbands A, Rodgerson MJ, Dowell J, Patterson F. Evaluating the validity of an integrity-based situational judgement test for medical school admissions. BMC Med Educ. 2015;15:144. doi: 10.1186/s12909-015-0424-0 PMID: 26330210.
7. Kiessling C, Bauer J, Gartmeier M, Iblher P, Karsten G, Kiesewetter J, et al. Development and validation of a computer-based situational judgement test to assess medical students' communication skills in the field of shared decision making. Patient Educ Couns. 2016;99(11):1858–64. doi: 10.1016/j.pec.2016.06.006 PMID: 27345253.
8. Kepes S, Keener SK, Lievens F, McDaniel MA. An integrative, systematic review of the situational judgment test literature. J of Manag. 2025;51:2278–319. doi: 10.1177/01492063241288545.
9. Koeppen K, Hartig J, Klieme E, Leutner D. Current issues in competence modeling and assessment. Z Psychol. 2008;216:61–73. doi: 10.1027/0044-3409.216.2.61.
10. Weinter FE. Concept of competence: a conceptual clarification. In: Rychen DS, Salganik LH, editors. Defining and selecting key competencies. Seattle: Hogrefe; 2001. pp. 45–65.
11. Blömeke S, Gustafsson J-E, Shavelson RJ. Beyond dichotomies. Z Psychol. 2015;223(1):3–13. doi: 10.1027/2151-2604/a000194.
12. Tabachnick BG, Fidell LS. Using multivariate statistics. 6th edition. New York: Pearson; 2013.
13. Kline RB. Principles and practice of structural equation modeling. 4th edition. New York: The Guilford Press; 2016.
14. Brown TA. Confirmatory factor analysis for applied research. 2nd edition. New York: The Guilford Press; 2015.
15. Furr RM. Psychometrics: an introduction. 4th edition. California: SAGE; 2022.
16. Raykov T, Marcoulides GA. Introduction to psychometric theory. New York: Routledge; 2011.
17. Stevens J. Applied multivariate statistics for the social sciences. 5th edition. New York: Routledge; 2009.
18. Hair JF, Black WC, Babin BJ, Anderson RE. Multivariate data analysis. 8th edition. Hampshire: Cengage; 2018.
19. Fabrigar LR, Wegener DT, MacCallum RC, Strahan EJ. Evaluating the use of exploratory factor analysis in psychological research. Psychol Methods. 1999;4(3):272–99. doi: 10.1037/1082-989X.4.3.272.
20. Hachmeister C-D. [What do women study? What do men study? – Students and first-year students by gender. CHE DataCHECK] [Internet]. 2025 Mar [cited 2025 Aug 26]. Available from: https://hochschuldaten.che.de/was-studieren-frauen-was-studieren-maenner/. German.
21. Kurtz S, Silverman J, Benson J, Draper J. Marrying content and process in clinical method teaching: enhancing the Calgary-Cambridge Guides. Acad Med. 2003;78(8):802–9. doi: 10.1097/00001888-200308000-00011. PMID: 12915371.
22. Frank JR, Danoff D. The CanMEDS initiative: implementing an outcomes-based framework of physician competencies. Med Teach. 2007;29(7):642–7. doi: 10.1080/01421590701746983 PMID: 18236250.
23. Whetzel DL, Sullivan TS, McCloy RA. Situational judgment tests: an overview of development practices and psychometric characteristics. PAD. 2020;6(1):1–16. doi: 10.25035/pad.2020.01.001.
